# Giant Osteoma of Zygoma Mimicking Pseudo-Temporo-Mandibular Joint Ankylosis: A Case Report

**DOI:** 10.7759/cureus.29020

**Published:** 2022-09-11

**Authors:** Bhushan P Mundada, Nitin D Bhola, Apoorva Mishra, Pawan Hingnikar, Prachet Dakshinkar, Prafulla Gaikwad

**Affiliations:** 1 Oral and Maxillofacial Surgery, Sharad Pawar Dental College and Hospital, Datta Meghe Institute of Medical Sciences, Wardha, IND

**Keywords:** tmj ankylosis, trismus, reduced mouth opening, osteoma, pseudo-tmj ankylosis

## Abstract

Giant osteoma is a rare entity in the head and neck region when compared to long bones. Even in the head and neck region, the paranasal sinuses are commonly associated, but the involvement of jaw bones is very rare. The lesions are usually asymptomatic and so remain undiagnosed for years. In the reported case, the distinct presentation with reduced mouth opening made it more confusing to diagnose as it became somewhat similar to symptoms of temporo-mandibular joint disorder. The involvement of the zygomatic bone with its extension into the mandibular ramus region made it more unique in its presentation. The objective of the current article is to present an unusual case of giant osteoma of zygoma causing reduced mouth opening, misdiagnosed as a true intra-articular temporo-mandibular joint ankylosis previously. This was then diagnosed correctly with help of a computed tomography scan and histopathology and treated with surgical excision.

## Introduction

Exostosis/osteoma is a benign overgrowth from a normal bone and is broadly classified as central, peripheral, and extra-skeletal types [1,2]. Osteomas are commonly associated with long bones and are extremely rare in the head and neck region. Though, if present, the most commonly associated sites are the paranasal sinuses and mandible [3]. It is usually asymptomatic exhibiting continuous growth during adulthood and thus can be detected incidentally on radiographic examination. Occasionally a patient may present with striking features like facial asymmetry or severe dysfunction, depending upon the sites involved, and thus osteoma should be considered as one of the differential diagnoses [4].

We present an unusual case of a giant osteoma of zygoma extending to the mandibular ramus region. This led to the reduction in mouth opening (MO) mimicking pseudo-temporo-mandibular joint (TMJ) ankylosis, which was repeatedly misdiagnosed as true TMJ ankylosis. The most unique feature of the present case scenario was its unique site and extent of involvement which hindered the MO thereby depicting false clinical features and adding to the confusion. This case report aims to envisage surgeons about diagnostic ambiguity in such cases and their appropriate treatment. 

## Case presentation

**Figure 1:**
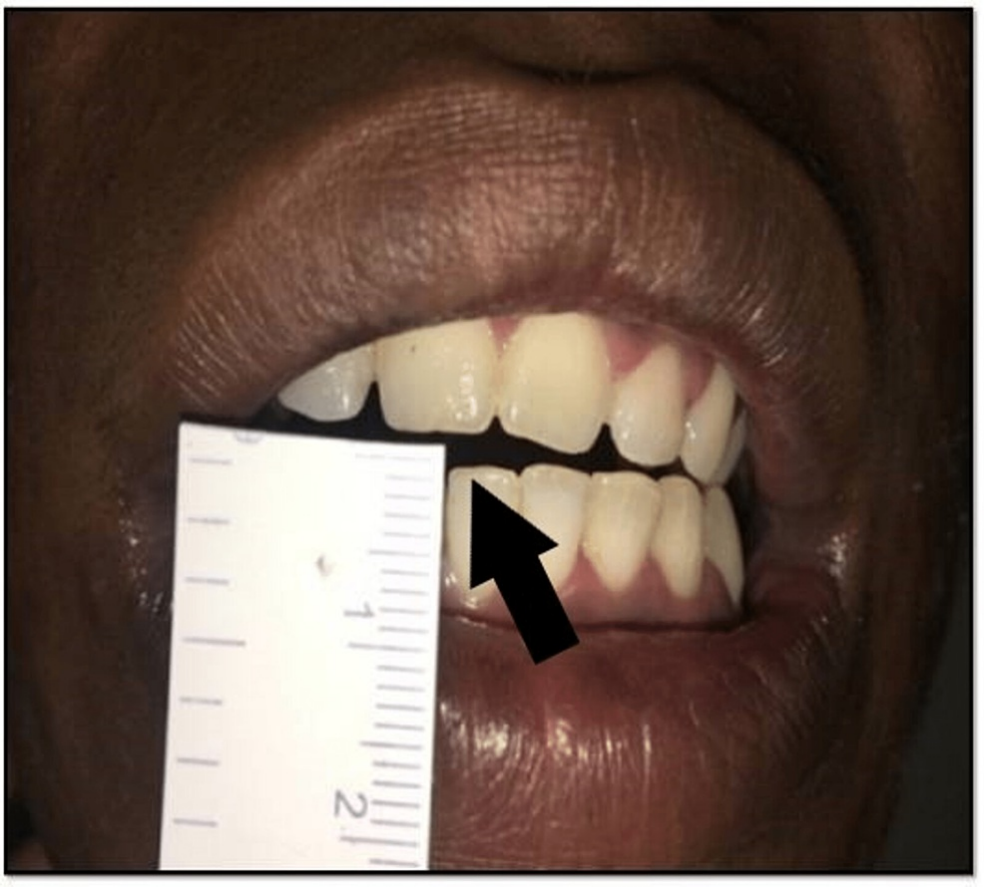
Clinical picture showing reduced mouth opening

Since seven years, a patient aged 19 years presented with the complaint of progressive reduction in MO, along with a noticeable gradual increase in the bony hard mass on the left side of the face, with no previous incidence of any associated trauma and infection to the Out-Patient Unit of Maxillofacial Surgery. Previously misdiagnosed as fibrous TMJ ankylosis owing to the patient’s symptoms and cone beam computed tomography (CBCT), brisement force, i.e. forceful MO, was done under sedation at a private clinic. The patient attained no symptomatic relief and was referred to DMIMS, Sawangi, Meghe, for further management. On preliminary examination, inter-incisal MO was 2-3 mm, and restricted TMJ movements were present along with deviation on the left side (Figure 1). Bony hard mass was palpable on the left zygoma region extending toward the mandible mimicking ossification in masseter muscle or exostosis.

**Figure 2:**
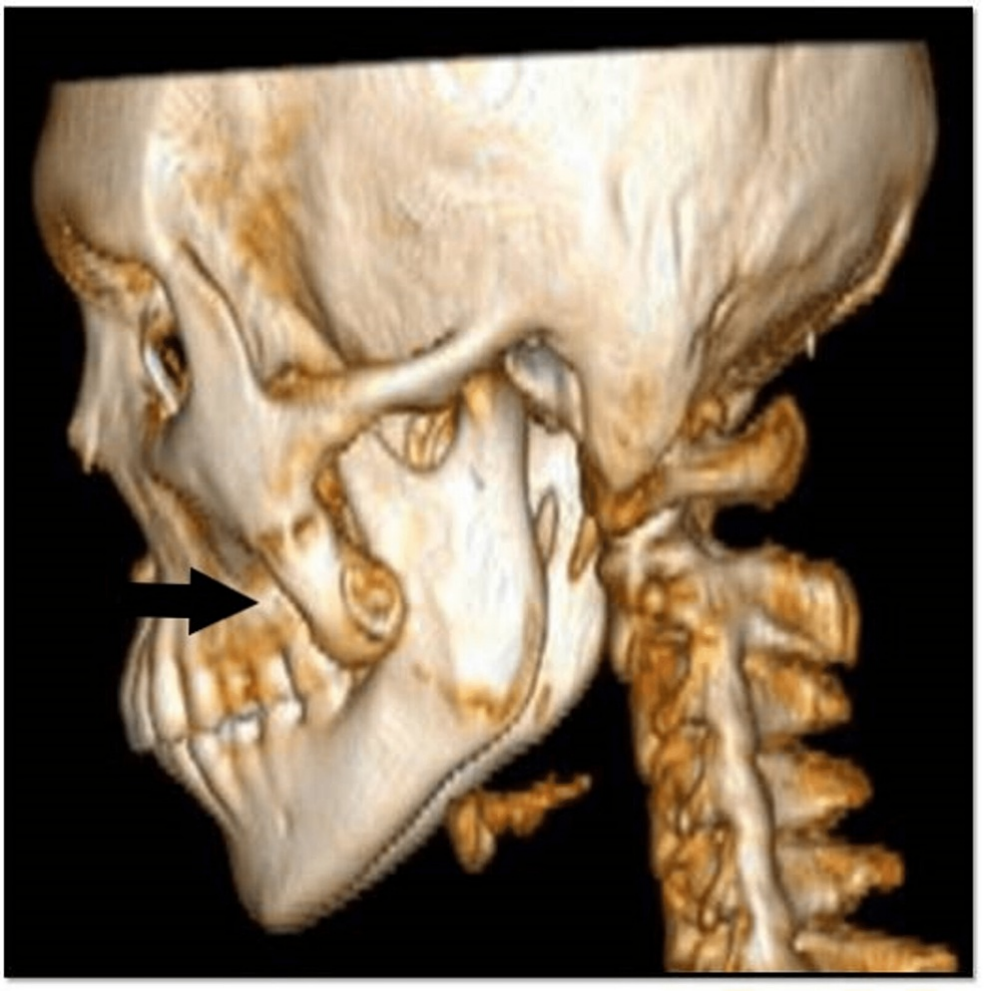
Cone beam computed tomography showing unusual bony growth anterior to coronoid process

**Figure 3:**
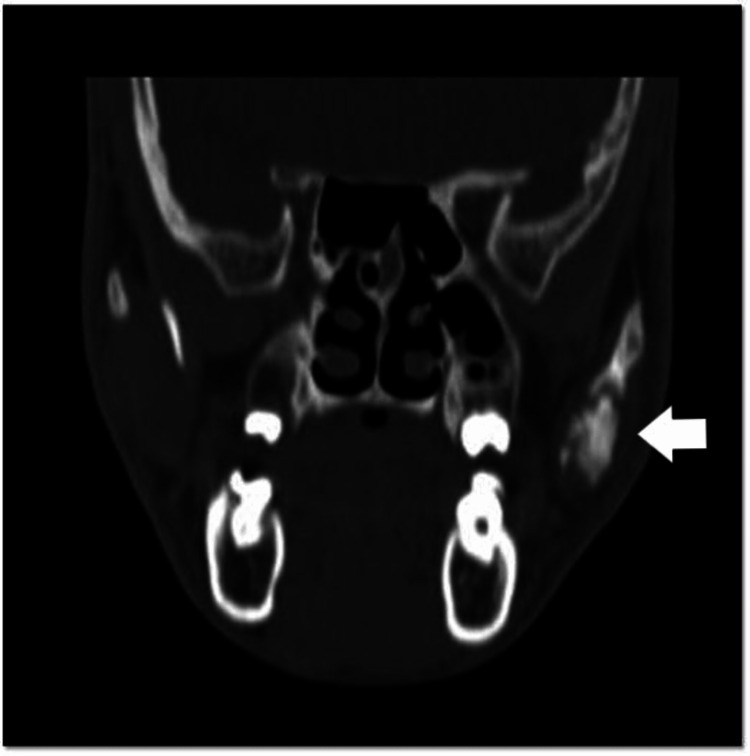
Computed tomography showing bony mass extending from zygoma to ramus of mandible

CBCT (Figure 2) showed bony overgrowth extending from zygoma to mandible but due to lack of essential cuts repeat computed tomography (CT) scan was advised. CT scan showed bony outgrowth of 3.2 x 1.4 cm approximately extending from the outer cortex of mandibular ramus to left zygomaticomaxillary sutures (Figure 3), suggestive of an exostosis, causing pseudo-ankylosis.

**Figure 4:**
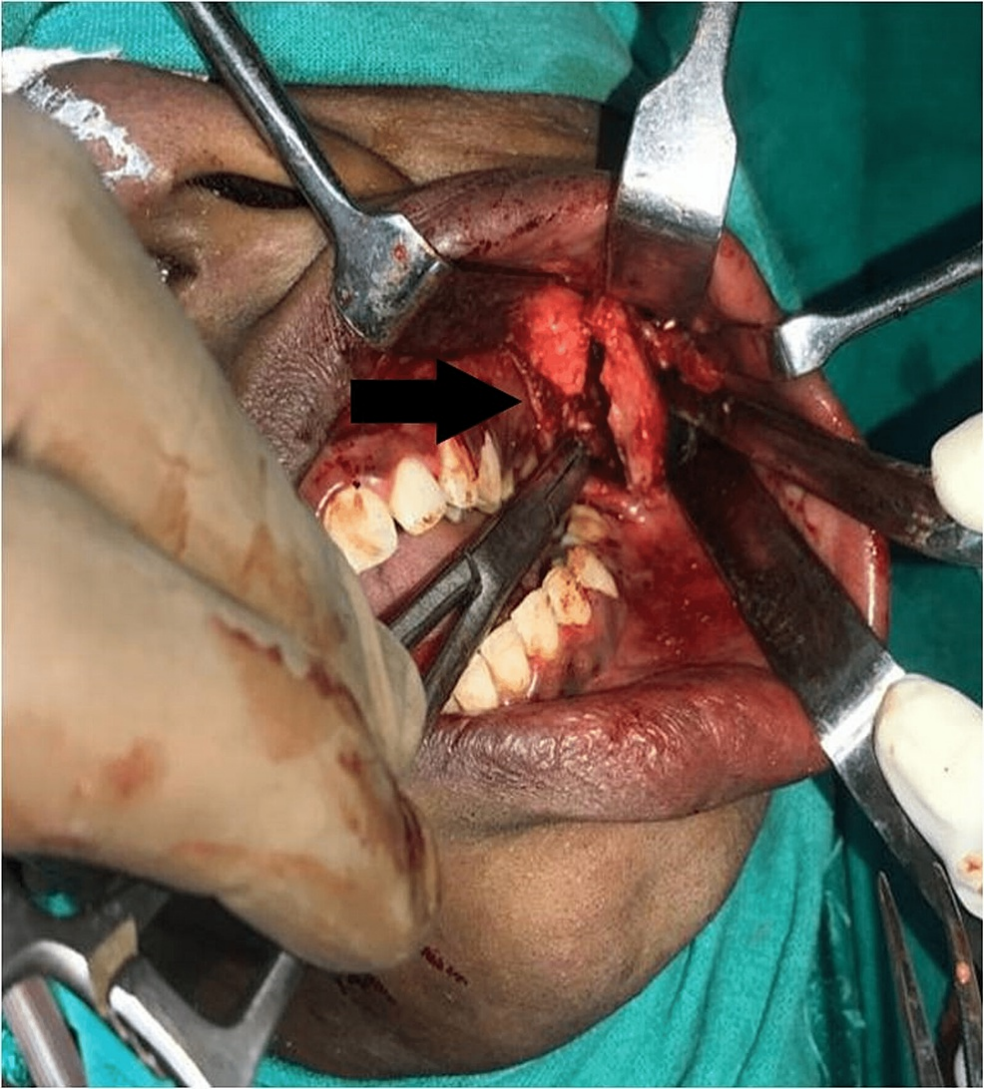
Intraoperative picture showing bony mass

After obtaining pre-anesthetic fitness and the patient’s consent, exostosis was removed surgically under general anesthesia, by intra-oral approach. The marking of the incision commenced from the retromolar region extending up to the anterior border of the ramus giving a slight curve into the maxillary vestibular region just short of the opening of the parotid duct. Preserving all vital structures, a full-thickness mucoperiosteal flap was raised to approach the tumor (Figure 4).

**Figure 5:**
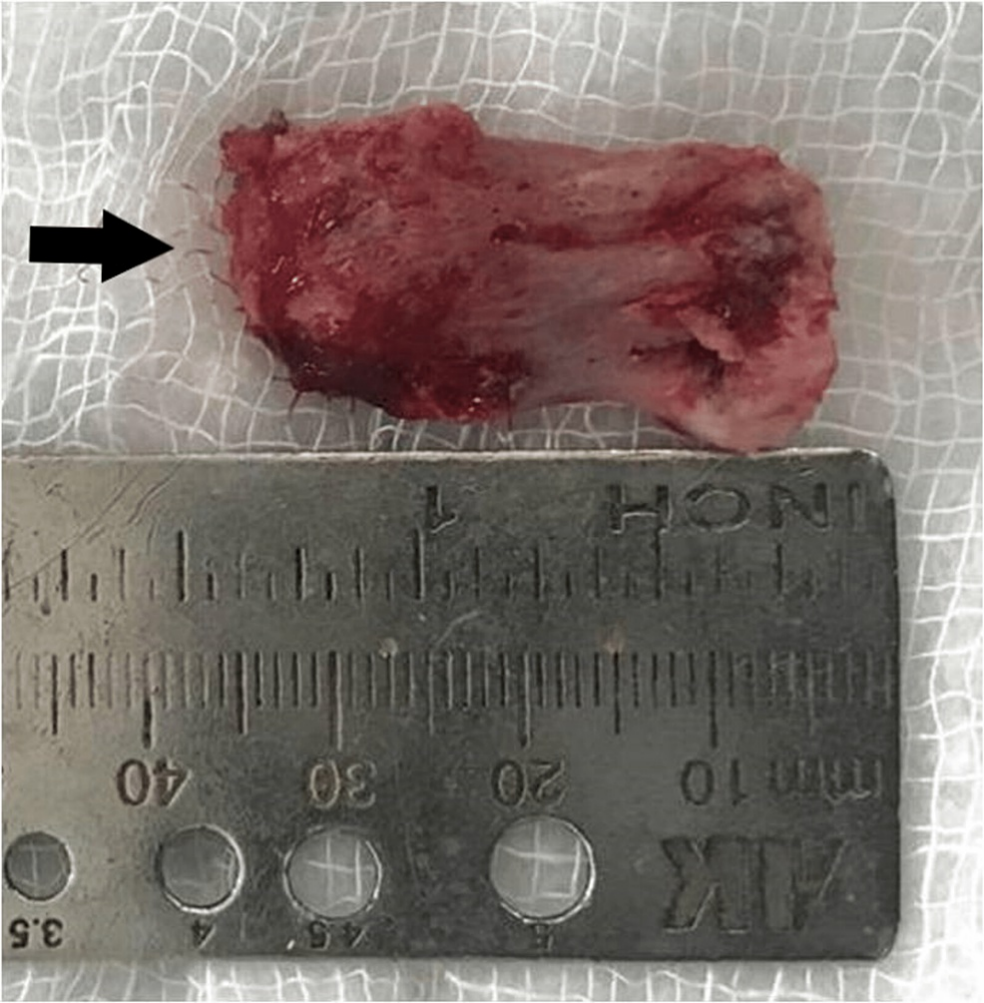
Picture showing excised tumor mass

**Figure 6:**
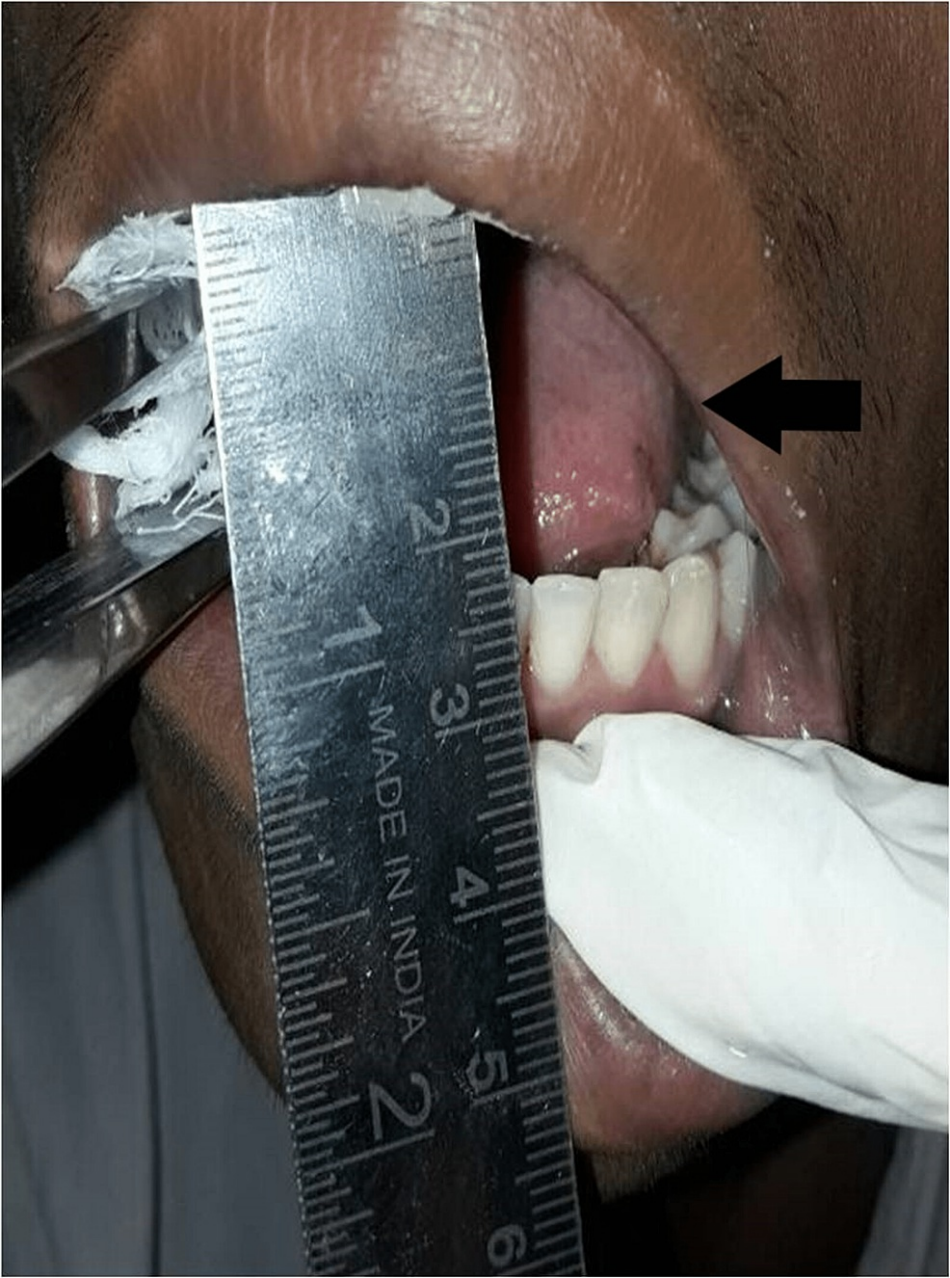
Immediate postoperative mouth opening

After achieving adequate exposure, the extent of the involved bone with a few millimeters of normal adjacent bone was demarcated with 701 round bur. The tumor was excised completely followed by contouring of residual bone (Figure 5). Intraoperative MO amounting to 35 mm was achieved, which was maintained by physiotherapy postoperatively (Figure 6).

The patient was kept on long-term regular follow-up for four years, along with vigorous MO exercises. Histopathological examination reported the presence of osteoid tissue along with fibro-cellular marrow tissue establishing the final diagnosis as osteoma.

## Discussion

Osteomas are generally known to be benign in their characteristics and have a possible origin from the multiplication of a lamellar bone, cancellous bone, or a combination [5]. They can be central or peripheral in origin and can be present extra-skeletally, having derivations from the internal part of the bone, and periosteum [1,2]. Etiology is unknown but few authors suggest that it could be genetic, due to environmental factors, masticatory hyperfunction, or continued jaw bone growth [6]. In the present case, there is no history of associated trauma, syndromic features, or any known etiological factors, thus giving a predilection of developmental etiology. Incidences of osteomas are more in males than females (2:1) [5]. They commonly mature in the adolescent years and steadily progress during the course of successive years. The most frequent incidence numbers are 29.4 to 40.5 years in the maxillofacial region [1].

In the maxillofacial region, osteomas have been documented in sinuses, temporal and orbital bone, the external auditory canal (EAC), orbit, pterygoid plates, and rarely in jaw bones. Literature reports less than 1% of cases in the jaws [7]. In the mandible, osteomas were reported in the anterior, body, angle, and condyle region, ascending ramus, coronoid process, and sigmoid notch [3]. In the literature, there are several cases with an osteoma of the zygoma and zygomatic arch but none of the cases reported giant osteoma in the zygoma region extending to the mandible hindering the mandibular movements causing extra-articular, i.e. pseudo-TMJ, ankylosis. Literature reveals no consensus on the etiological factors. Various etiological factors like inflammation, stress during mastication, genetic predilection, environmental influences, or the presence of any systemic disease might be one of the reasons for osteomas [8].

Most commonly osteomas are asymptomatic and are diagnosed incidentally. However, in the present case, it has interfered with normal functioning and caused facial deformity. Reported symptoms were headache, exophthalmos, difficulty in eating, mandibular deviation on opening, and bone pain [1,9].

The differential diagnosis for the present case was a) ossification in masseter (myositis ossificans), b) osteochondroma of the coronoid process (Jacob’s disease), and c) ossifying fibroma or condensing osteitis. Myositis ossificans is a rare heterotopic bone formation within a muscle, with the masseter being the most frequently affected, mostly preceded by traumatic injuries. Osteochondroma of the coronoid process also has similar features but it forms a pseudo joint with the inner surface of the zygoma and is capped with cartilage. Ossifying fibroma belongs to the group of fibro-osseous lesions affecting both the maxillary and mandibular bones. It comprises differential calcified tissue mimicking either bone, cementum, or both. Condensing osteitis reflects impaired bone growth wherein bone arrangement is elicited by the milder infections of the dental pulp. 

Depending on the location, conventional regional radiographs and histopathological examinations are enough to diagnose osteomas and differentiate the above-mentioned conditions but for proper surgical planning, a CT scan or CBCT is recommended. Due to the lack of reported incidence, as in the current situation, such cases are misdiagnosed as TMJ disorders and treated accordingly. TMJ disorders are also associated with similar symptoms along with severe pain. In the present case, deviation on the left side, reduced inter-incisal MO, and bony growth were seen, and CT findings provisionally diagnosed it as osteoma which was confirmed by histopathology.

Osteomas are normally self-limiting and no treatment is required. Although, sometimes they may overgrow and show adverse symptoms as mentioned above which mandates surgical removal or shaving of bony mass. Gadre et al. have reported a case of mandibular body region wherein Piezo surgical removal of the tumor was done to preserve the vital structures [10]. Recurrence and malignant transformation of such lesions are very rare [1]. However, the patient is still an adolescent and is still in his growing phase, and thus was kept on regular follow-up and vigorous MO exercises were advised.

Thus, “osteoma with such location can be similar to TMJ ankylosis in clinical presentations, but radiographic appearances of the two conditions are characteristic. Identification of these radiographic features, preferably in CT or CBCT, is helpful to prevent misdiagnosis. We also suggest that the osteoma in our case is of developmental origin in the absence of other relevant causes.”

## Conclusions

The authors suggest that clinicians should not be prejudiced against a particular diagnosis, and differential diagnoses should be ruled out. In the present case, earlier, clinicians misdiagnosed it as a TMJ disorder and focused the radiographic investigation and treatment on its line. Overall attentive clinical examination and CT scan will serve as a better modality in the presence of various differential diagnoses in the present case scenario.

## References

[1] Longo F, Califano L, De Maria G, Ciccarelli R (2001). Solitary osteoma of the mandibular ramus: report of a case. J Oral Maxillofac Surg.

[2] Johann AC, de Freitas JB, de Aguiar MC, de Araújo NS, Mesquita RA (2005). Peripheral osteoma of the mandible: case report and review of the literature. J Craniomaxillofac Surg.

[3] Durão AR, Chilvarquer I, Hayek JE, Provenzano M, Kendall MR (2012). Osteoma of the zygomatic arch and mandible: report of two cases. Rev Port Estomatol Med Dent Cir Maxilofac.

[4] Bartoli MM, Maciel LF, de Alencar MG, da Silva TC, Vasconcellos RJ (2018). Surgical treatment of osteoma in the basilar region of the mandible. J Craniofac Surg.

[5] Kerckhaert A, Wolvius E, van der Wal K, Oosterhuis JW (2005). A giant osteoma of the mandible: case report. J Craniomaxillofac Surg.

[6] Shamim T (2017). Bilateral maxillary and mandibular buccal exostosis: a self reported case and a proposal to include buccal exostosis under miscellaneous disorders of revised working classification of the psychosomatic disorders pertaining to dental practice. Korean J Pain.

[7] Matthies L, Rolvien T, Pakusa TJ (2019). Osteoid osteoma of the mandible - clinical and histological findings. Anticancer Res.

[8] Alkurt MT, Peker I, Demirel O, Akay G, Gungor K, Ucok O (2016). The prevalence of antral exostoses in the maxillary sinuses, evaluated by cone-beam computed tomography. J Dent Sci.

[9] Mittal A, Iyer N (2008). Large peripheral osteoma of the mandible. Oral Radiol.

[10] Gadre P, Singh D, Gadre K, Khan I (2016). Piezosurgery for excision of large osteoid osteoma. J Craniofac Surg.

